# UV light absorption parameters of the pathobiologically implicated bilirubin oxidation products, MVM, BOX A, and BOX B

**DOI:** 10.1016/j.dib.2018.04.010

**Published:** 2018-04-12

**Authors:** Nathaniel A. Harris, Robert M. Rapoport, Mario Zuccarello, John E. Maggio

**Affiliations:** a Department of Pharmacology and Cell Biophysics, College of Medicine, University of Cincinnati, Cincinnati, OH, USA; b Research Service, Veterans Affairs Medical Center, Cincinnati, OH, USA; c Department of Neurosurgery, College of Medicine, University of Cincinnati, Cincinnati, OH, USA; d Surgical Service, Veterans Affairs Medical Center, Cincinnati, OH, USA

**Keywords:** Bilirubin oxidation products, BOX A, BOX B, MVM, λ_max_, Extinction coefficient, Synthesis

## Abstract

The formation of the bilirubin oxidation products (BOXes), BOX A ([4-methyl-5-oxo-3-vinyl-(1,5-dihydropyrrol-2-ylidene)acetamide]) and BOX B (3-methyl-5-oxo-4-vinyl-(1,5-dihydropyrrol-2-ylidene)acetamide), as well as MVM (4-methyl-3-vinylmaleimide) were synthesized by oxidation of bilirubin with H_2_O_2_ without and with FeCl_3_, respectively. Compound identity was confirmed with NMR and mass spectrometry (MS; less than 1 ppm, tandem MS up to MS^4^). UV absorption profiles, including λ_max_, and extinction coefficient (ε; estimated using NMR) for BOX A, BOX B, and MVM in H_2_O, 15% CH_3_CN plus 10 mM CF_3_CO_2_H, CH_3_CN, CHCl_3_, CH_2_Cl_2_, and 0.9% NaCl were determined. At longer wavelengths, λ_max_'s for 1) BOX A were little affected by the solvent, ranging from 295–297 nm; 2) BOX B, less polar solvent yielded λ_max_'s of lower wavelength, with values ranging from 308–313 nm, and 3) MVM, less polar solvent yielded λ_max_'s of higher wavelength, with values ranging from 318–327 nm. Estimated ε’s for BOX A and BOX B were approximately 5- to 10-fold greater than for MVM.

**Specifications Table**

**Table:** 

Subject area	*Chemistry*
More specific subject area	*Bilirubin oxidation products detection*
Type of data	*Table, figure*
How data was acquired	*NMR, mass spectroscopy, UV spectrometry, HPLC*
Data format	*Raw, analyzed*
Experimental factors	*Oxidation of bilirubin, extraction with chloroform*
Experimental features	*Bilirubin oxidation products BOX A, BOX B, and MVM were synthesized by the oxidation of bilirubin, purified by HPLC and UV absorption profiles and extinction coefficients determined*
Data source location	*Cincinnati, OH USA*
Data accessibility	*The data are accessible within the article*.

**Value of the data**

• First report (to our knowledge) of UV absorption profile, including λ_max_, of MVM in solvents relevant to detection in biologic/pathobiologic samples.
• Comparison of UV absorption profiles of MVM with BOX A and BOX B.
• First report (to our knowledge) of BOX B extinction coefficient (ε; estimated using NMR), along with comparison to BOX A and MVM estimated ε’s in different solvents, along with MS at less than 1 ppm and tandem MS up to MS^4^.
• Novel methodology to increase MVM yield through FeCl_3_ inclusion in oxidation reaction mixture.
• Data will potentially assist in the detection and determination of these BOXes in pathobiologies associated with elevated bilirubin.

## 1 Data

The bilirubin oxidation products (BOXes), MVM (4-methyl-3-vinylmaleimide), along with BOX A ([4-methyl-5-oxo-3-vinyl-(1,5-dihydropyrrol-2-ylidene)acetamide]) and BOX B (3-methyl-5-oxo-4-vinyl-(1,5-dihydropyrrol-2-ylidene)acetamide), have been implicated in the deleterious effects associated with subarachnoid hemorrhage (SAH; [1], [2], [3], [4], [5]). The detection method utilized to determine the presence of these compounds is UV absorption associated with reversed phase-HPLC [1]. However, reports (to our knowledge) of the UV absorption profile and/or λ_max_ of MVM have not been reported for the solvent utilized in their detection (H_2_O/CH_3_CN), but are limited to CH_3_OH [6], [7]. Also, reports of these absorption characteristics are limited (to our knowledge) for BOX A to H_2_O and CH_3_CN, and for BOX B to H_2_O [1], [8]. Further, extinction coefficients (ε) for MVM and BOX A are limited (to our knowledge) to CH_3_OH and CH_3_CN, respectively [6], [7], [9], and are lacking for BOX B. Thus, it is anticipated that the present data will assist in the detection and quantitative determination of BOXes levels in biologic samples from SAH, as well as in other pathobiologies associated with elevated bilirubin.

### 1.1 UV absorption

**Fig. 1:**
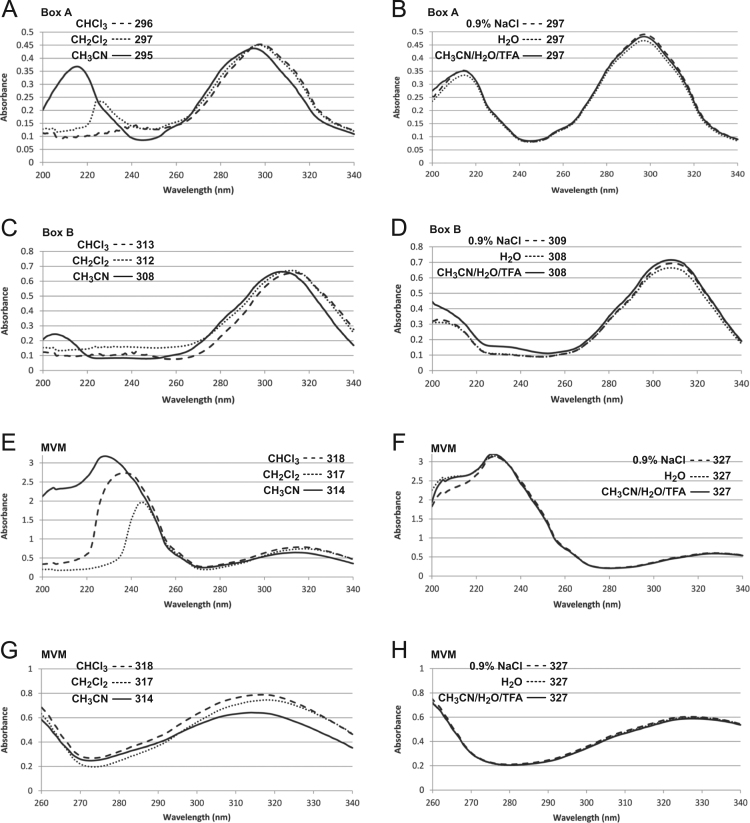
*Absorption spectra for BoxA, BoxB, and MVM*. Spectra were determined for Box A (A,B), Box B (C,D,) and MVM (E-H). In A,C,E, and G: CHCl_3_ (---), CH_2_Cl_2_ (^**…**^), and CH_3_CN (^___^). In B, D, F, and H: 0.9% NaCl (^__^), H_2_O (^**…**^) and 15% CH_3_CN/85% H_2_O with 10 mM CF_3_CO_2_H (TFA; (---)).

UV absorption spectra of BOX A, BOX B and MVM were determined in CHCl_3_, CH_2_Cl_2_, CH_3_CN, 15% CH_3_CN plus 10 mM CF_3_CO_2_H, H_2_O, and 0.9% NaCl (Fig. 1, Table 1). At longer wavelengths, BOX A λ_max_'s were little affected by the solvent, ranging from 295–297 nm (Fig. 1, Table 1). With BOX B, less polar solvent yielded λ_max_'s of lower wavelength, with values ranging from 308–313 nm (Fig. 1, Table 1). With MVM, less polar solvent yielded λ_max_'s of higher wavelength, with values ranging from 318–327 nm (Fig. 1, Table 1). These λ_max_ values corresponded to previously reported λ_max_'s at longer wavelengths, as limited to the following solvents: BOX A of 300 nm in H_2_O and 295 nm in CH_3_CN [1], [2], BOX B of 310 nm in H_2_O [1], and MVM of 317 and 319 nm in CH_3_OH [6], [7].

**Table 1:** Solvent Effects on λ_max_^a^ and ε^b^.

**Solvent**	**Box A**		**Box B**		**MVM**	
	**λ**_**max**_	**ε**	**λ**_**max**_	**ε**	**λ**_**max**_	**ε**
CHCl_3_	296	13,000	313	24,200	318	2660
CH_2_Cl_2_	297	12,200	312	24,300	317	2820
CH_3_CN	295	10,600	308	22,200	314	2290
CH_3_CN (15%) + TFA (10 mM)	297	11,900	309	22,400	327	2150
H_2_O	297	11,000	308	19,000	327	2130
NaCl (0.9%)	297	11,600	308	21,000	327	2100

a nm

b L/mol•cm

### 1.2 Extinction coefficients (ε)

Calculated ε’s for BOX A, BOX B, and MVM at their respective λ_max_'s in CHCl_3_, CH_2_Cl_2_, CH_3_CN, 15% CH_3_CN plus 10 mM CF_3_CO_2_H, H_2_O, and 0.9% NaCl, ranged from 10,600-13,000, 19,000–24,200, and 2,100-2,820 L/mol-cm respectively (Table 1). The ε determined using the actual amount of *Z*-BOX A (complete chemical synthesis), at λ_max_ 295 in CH_3_CN, was 17,000 L/mol-cm [9]. Thus, the present Box A ε likely represents a low estimate (Table 1). The estimated MVM ε (Table 1) is similar to that reported for MVM at λ_max_ 317 and 319 nm in CH_3_OH of 2,300 and 2,290 L/mol-cm [6], [7].

## 2 Experimental design, materials and methods

### 2.1 Synthesis

Bilirubin solubilization was performed at room temperature in an aluminum foil wrapped vessel due to the reported light sensitivity of BOX A, BOX B, and MVM [1], [8], [10]. One or more 50 mg portions of bilirubin were incubated in 25 ml 0.2 M NaOH(aq) with occasional vortexing over 24–72 h [1], [10]. The dark red bilirubin solution was then buffered by addition of 7.5 ml of 0.5 M Tris base before neutralization with 0.4 ml of 12.3 M HCl(aq) to pH 7.0. Overtitration of the dark red solution to lower pH resulted in a green solution. The neutralized (pH 7) buffered bilirubin solution was immediately used for oxidation with H_2_O_2_. With prolonged storage, bilirubin precipitated from this supersaturated solution.

As performed under dim ambient light and in an unlit fume hood (and with dim ambient light) the neutral buffered solution (now in 0.1 M TrisHCl, pH 7.0, 0.15 M NaCl) was oxidized for 24 h with 8% H_2_O_2_ (final concentration). For MVM synthesis, 0.5 M FeCl_3_ was added (novel procedure) to the bilirubin solution prior to H_2_O_2_ and the oxidation allowed to proceed for 10 min. Each aqueous reaction mixture (about 45 ml per 50 mg bilirubin) was extracted twice with 6 ml CHCl_3_ or CH_2_Cl_2_ (recoveries of BOX A, BOX B, and MVM were similar with CHCl_3_ and CH_2_Cl_2_) and the combined organic phase extracted once with 1 ml water, evaporated to ~2 ml at <50 °C and atmospheric pressure, transferred to microfuge tubes, and evaporated to near dryness. Additional ~2 ml aliquots of extract were repeatedly added, each followed by evaporation to near dryness. The final addition of washed extract was evaporated to dryness and reconstituted in 1 ml 1% CH_3_CN(aq) for purification by reversed phase (RP)-HPLC.

### 2.2 Purification by RP-HPLC

RP-HPLC (0.1 cm light path; Shimadzu LC-10AT, Shimadzu Scientific Instruments, Columbia, MD) was used for both purification and analysis of the bilirubin oxidation products. As performed under dim light, organic solvent extracts of BOX A and BOX B, as well as MVM, reconstituted in 1% CH_3_CN(aq) were diluted as necessary into the RP-HPLC starting buffer of 2% CH_3_CN:98% H_2_O (v:v) containing 10 mM M CF_3_CO_2_H. Injections of 1.0–1.5 ml were made onto a Vydac 218TP C-18 5 µm column (250 × 4.6 mm) with guard column equilibrated with 2% CH_3_CN containing 0.01 M CF_3_CO_2_H. The guard column was necessitated by the detection of a small amount of residual H_2_O_2_ in the CHCl_3_ and CH_2_Cl_2_ extracts of the bilirubin-H_2_O_2_ reaction mixtures. An attempt to remove the H_2_O_2_ with CH_3_CH_2_OH (molar CH_3_CH_2_OH:H_2_O_2_ ratio 1.5:1) addition to the reaction mixtures actually caused a 10-fold increase in the amount of substrate detected as H_2_O_2_. H_2_O_2_ was not detected in RP-HPLC-purified oxidation products.

The column was eluted (1 ml/min) with a continuous gradient of 0.5% CH_3_CN/min (2% to 18% CH_3_CN) over 32 min, followed by steeper gradients and higher CH_3_CN concentration for washing the system between runs. Eluates were monitored from 210–350 nm using a diode array spectrophotometer and flow cell and were collected in aluminum foil wrapped test tubes.

**Fig. 2:**
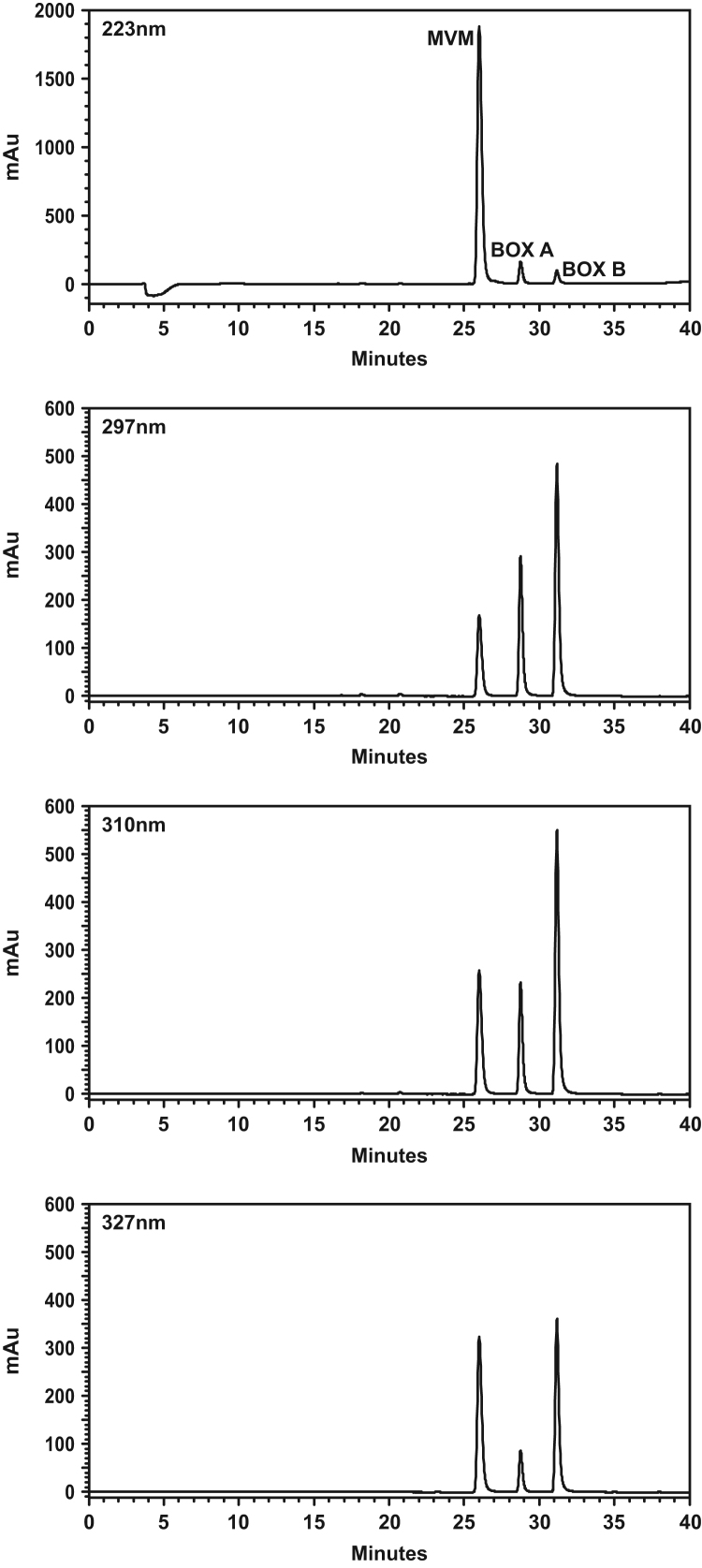
*HPLC traces of BOX A, BOX B, and MVM*. Standards were prepared and subjected to RP-HPLC (Materials and Methods). Absorption was determined at 223 nm (upper trace) and at the respective λ_max_'s of BOX A, BOX B, and MVM, which were 297 (second most upper trace), 310 (third most upper trace), and 327 nm (lower trace), respectively (see Fig. 1 and Table 1).

RP-HPLC of the combined products of bilirubin-H_2_O_2_ reaction mixtures with and without Fe^3+^ yielded three peaks with retention times at 26.0, 28.7, and 31.2 min, respectively (Fig. 2). These retention times corresponded to eluting CH_3_CN concentrations of 12.8, 14.4, and 15.6% (v/v), respectively. UV absorption at other retention times was not detected at 297, 310, and 327 nm, *i.e*., at the longer wavelength λ_max_'s of the compounds with 26.0, 28.7, and 31.2 retention times, respectively, as well as at 223 nm (Fig. 1, Fig. 2; Table 1), indicative of a purified preparation. This relative order of retention time of MVM, BOX A, and BOX B differs from that which a laboratory previously reported, which was BOX A, BOX B, and then MVM [1], [5]. While this difference in relative order of retention time may be due to differences in column properties, it should also be considered that the present inclusion of CF_3_CO_2_H in the solvent resulted in ion pairing with BOX A and BOX B (retention times of the ion pairs would be increased as compared to the non-paired species).

From the bilirubin-H_2_O_2_ oxidation in the absence of Fe^3+^, the ratio of MVM:BOX A:BOX B (BOX A set at absorption unity) formed at their respective λ_max_'s (Fig. 1, Fig. 2, Table 1) was 0.10 ± 0.03:1.0:0.95 ± 0.05, respectively (mean ± SE; n = 5). Several minor peaks were also observed (determined prior to further RP-HPLC purification). Incubation at times shorter or longer than 24 h did not result in additional MVM formation. Yields after purification of BOX A and BOX B were ~1% each, based on starting material and measured by UV spectroscopy (calculated with ε’s as described below; Table 1).

From the bilirubin-H_2_O_2_ oxidation in the presence of Fe^3+^, the ratio of MVM:BOX A:BOX B (MVM set at absorption unity) formed at their respective λ_max_'s (Fig. 1, Fig. 2, Table 1) was 1.0:0.05 ± 0.01:0.04 ± 0.01, respectively (mean ± SE; n = 5). Several minor peaks were also observed (determined prior to further RP-HPLC purification). Incubation for 1, 5, 30, 45, and 60 min did not increase BOX A and BOX B formation while MVM formation was reduced. The reaction yielded ~5% MVM, based on starting material and measured by UV spectroscopy (calculated with ε; Table 1). Increased MVM formation with Fe^3+^ inclusion in the bilirubin-H_2_O_2_ reaction mixture is consistent with the dependency of MVM formation following H_2_O_2_ oxidation of ferriprotoporphyrin IX on the chelated iron [11] as well as the oxidation of bilirubin by CrO_3_ [6].

Present yields are generally consistent with earlier reports of <5% and 4% formation of BOX A, BOX B and MVM [1], [10]. While one of these reports [1] also demonstrated significant MVM synthesis (MVM:BOX A:BOX B = 2.8:1:0.9; determined at 320 nm and as presently calculated with BOX A set to unity), the increased MVM formation was possibly due to a somewhat greater H_2_O_2_ concentration in the reaction mixture with bilirubin (13% H_2_O_2_). On the other hand, highly variable amounts of MVM were formed by oxidation of bilirubin with ~10% H_2_O_2_ [10]. Hydrogen peroxide oxidation of biliverdin instead of bilirubin did not increase the yield of MVM.

### 2.3 Stability

After purification, BOX A, BOX B, and MVM samples shielded with aluminum foil from light were stable for at least 6 mo at −20 °C and for 24 h at room temperature in 14.6% CH_3_CN (eluting solvent), as determined by RP-HPLC; *i.e*., no loss of compound or detection of additional absorption peaks through the UV absorption spectrum. Removal of the aluminum foil and exposure of BOX A, BOX B, and MVM (in 14.6% CH_3_CN in a clear polypropylene microfuge tube) to ambient light for 24 h decreased recovery by 10%, 15%, and 5%, respectively, and the appearance of peaks at 18.3 min and 20.8 min with a ratio 1.13:1, and with λ_max_'s of 288 and 296 nm, respectively.

### 2.4 UV absorption spectrometry

UV spectra were performed in a SpectraMax M5 (Molecular Devices, Sunnyvale, CA, USA).

### 2.5 ^1^H-NMR

**Fig. 3:**
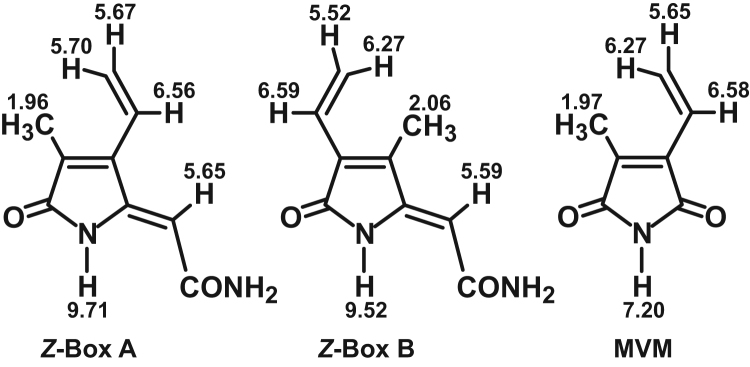
*Structures of BOX A, BOX B, and MVM and corresponding*^*1*^*H-NMR data.* Structures of BOX A and BOX B depicted as *Z* regio-isomers (after Seidel et al., 2015) and MVM with ^1^H-NMR data from Table 2.

For compound identification and ε determinations, analytic samples of BOX A, BOX B, and MVM (CHCl_3_ extraction) were loaded onto a C_18_ separation cartridge (Sep-Pak), washed with 1 ml D_2_O and eluted with 1.5 ml, 80% CD_3_CN (in D_2_O). Samples were then evaporated to dryness under N_2_ and reconstituted in 1 ml CD_3_CN. BOX A, BOX B, and MVM chemical shifts and coupling constants were determined on a DMX-500. Extinction coefficients (ε) at the respective λ_max_'s for BOX A, BOX B, and MVM were determined by titration in CH_3_OH (3.49 ppm singlet) and integration of signals relative to CH_3_OH under conditions of long recycle delay, and determination of UV absorption. ^1^H-NMR spectroscopy yielded chemical shifts and coupling constants for BOX A, BOX, B, and MVM consistent with previous reports (Fig. 3; Table 2; [1], [6], [9]).

**Table 2:** ^1^H-NMR chemical shifts and coupling constants for synthetic compounds^a^.

	**Box A**	**Box B**	**MVM**
**Chemical shifts**^b^			
δ vinyl-CH	6.56	6.59	6.58
δ vinyl-CH_2_ cis	5.70	6.27	6.27
δ vinyl-CH_2_ trans	5.67	5.52	5.65
δ -CH_3_	1.96	2.06	1.97
δ -CONHR	9.71	9.52	7.20
δ =CH-CONH_2_	5.65	5.59	n/a
**Coupling constants**^c^			
^3^J trans vinyl	17.9	17.9	17.8
^3^J cis vinyl	11.7	11.7	11.5
^2^J gem vinyl	<1.5	2.1	1.7

a Spectra collected at 500 MHz in CD_3_CN.

b Chemical shifts (δ) in ppm downfield of TMS, referenced to CHD_2_CN at 1.940 ppm.

c Coupling constants (J) in Hz.

### 2.6 MS

**Fig. 4:**
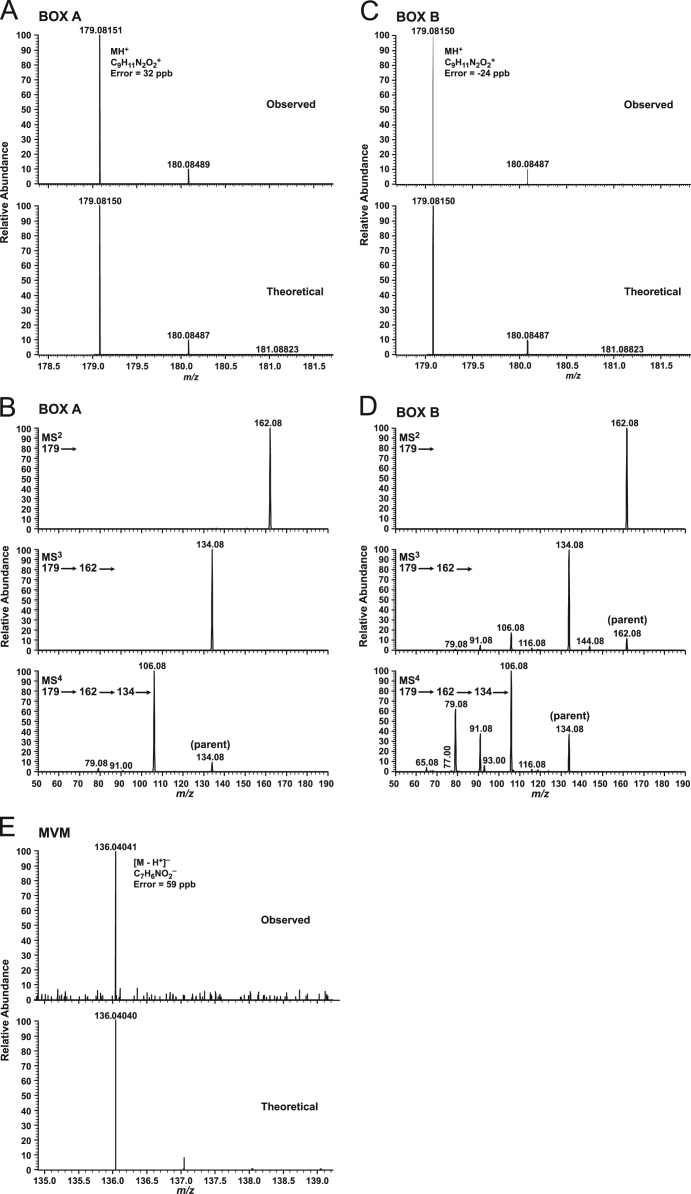
*Mass spectrometry (MS) of BOX A, BOX B, and MVM.* MS^n^ for BOX A (A and B), BOX B (C and D), and MVM (E). Corresponding group loss and error are indicated in Table 3.

Samples for MS were prepared by evaporation of compounds in aqueous CH_3_CN to dryness in an N_2_ stream at 40 °C, followed by reconstitution in 10% CH_3_CN/90% H_2_O containing 0.2% HCO_2_H. Lyophylization was avoided due to apparent loss of compounds. Samples obtained from RP-HPLC were infused into a Thermo Scientific LTQ-FT™ hybrid MS consisting of a linear ion trap and a Fourier transform ion cyclotron resonance (FT-ICR) MS. The standard electrospray ionization (ESI) source was operated in a profile mode for both positive and negative ions as indicated (Fig. 4, Table 3). The only possible elemental composition at 2 ppm mass error, but also even at 5 ppm, for 0–10 nitrogen, 0–15 oxygen, 0–30 carbons, and 0–60 hydrogens are those of BOX A and BOX B (as assessed for the positive ion mode), and for MVM (as assessed in both the positive and negative ion mode; Fig. 4, Table 3; consistent with 1,6,9,10). With MVM as the protonated molecular ion, the observed mass was *m/z* 138.05498 with a mass error of 180 ppb. For MVM, MS also suggested the apparent presence of the plastic antioxidant/stabilizer 1,10-bis(2,2,6,6-tetramethyl-4-piperidinyl-decanedioate), resulting from the (initial) carrying out of the FeCl_3_-bilirubin-H_2_O_2_ oxidation in a polypropylene vessel (“2. Experimental design, materials and methods; 2.1. Synthesis”; subsequent oxidations were performed in glass containers).

**Table 3:** FT-ICR.

	Product Ion	Error (ppb)	Loss
Box A			
MS^2^	162.05485	−649	NH_3_
MS^3^	134.05995	−675	CO
MS^4^	106.06507	−527	CO
Box B			
MS^2^	162.05485	−649	NH_3_
MS^3^	134.05997	−526	CO
	106.06506	−621	2CO
	91.05418	−515	[ion is C_7_H_7_^+^]
	144.04430	−628	H_2_O
	116.04940	−653	CO, H_2_O
MS^4^	106.06506	−621	CO
	79.05419	−344	[ion is C_6_H_7_^+^]
	91.05419	−445	[ion is C_7_H_7_^+^]
