# Supplementary material for: UV light absorption parameters of the pathobiologically implicated bilirubin oxidation products, MVM, BOX A, and BOX B
Source: Data Brief. 2018 Apr 12;18:1400–9. doi: 10.1016/j.dib.2018.04.010 (PMC5997574; doi:10.1016/j.dib.2018.04.010)
Supplement: Supplementary file 1 — Supplementary material [file mmc1.docx]

**Declaration of interest**

Conflicts of interest: none
